# LFE-UNet: A Lightweight Full-Encoder U-shaped Network for Efficient Semantic Segmentation in Medical Imaging

**DOI:** 10.2174/0115734056370555250426140155

**Published:** 2025-05-08

**Authors:** Qinghua Zhang, Yulei Hou, Changchun He, Zhengyu Zhai, Yunjiao Deng

**Affiliations:** 1 Department of Neurosurgery, Huazhong University of Science and Technology Union Shenzhen Hospital, Shenzhen 518052, China; 2 School of Mechanical Engineering, Dongguan University of Technology, Dongguan 523015, China; 3 School of Mechanical Engineering, Yanshan University, Qinhuangdao 066004, China; 4 The 6th Affiliated Hospital of Shenzhen University Health Science Center, Shenzhen 518060, China; 5 Biosmed Technology (Shenzhen) Co.Ltd., Shenzhen 518000, China

**Keywords:** Semantic segmentation, Full-encoder skip connections, Lightweight, U-Net, Medical imaging

## Abstract

**Background:**

Semantic segmentation algorithms are essential for identifying and segmenting human organs and lesions in medical images. However, as U-Net variants enhance segmentation accuracy, they often increase in parameter count, demanding more sophisticated and costly hardware for training.

**Objective:**

This study aims to introduce a lightweight U-Net that optimizes the trade-off between network parameters and segmentation accuracy, while fully leveraging the encoder's feature extraction capabilities.

**Methods:**

We propose a lightweight full-encoder U-shaped network, termed LFE-UNet, which employs full-encoder skip connections, encompassing all encoder layers. This model is designed with a reduced number of basic channels—specifically, 8 instead of the typical 64 or 32—to achieve a more efficient architecture.

**Results:**

The LFE-UNet, when integrated with ResNet34, achieved a *Dice* score of 0.97385 on the ISBI LiTS 2017 liver dataset. For the BraTS 2018 brain tumor dataset, it obtained 0.87510, 0.93759, 0.87301, and 0.81469 on average, WT, TC, and ET, respectively. The paper also discusses the impact of varying basic channel numbers *n* and encoder layer counts *N* on the network's parameter efficiency, as well as the model's robustness to different levels of Gaussian noise in images and salt and pepper noise in labels. Additionally, the influence of different loss functions is explored.

**Conclusion:**

The LFE-UNet proves that high segmentation accuracy can be attained with a markedly lower parameters, fully utilizing the full-scale encoder's feature extraction. It also highlights the significance of loss function selection and the effects of noise on segmentation accuracy.

## 1 INTRODUCTION

Deep learning advancements and increased computing power have made automatic medical image analysis a pivotal direction in smart healthcare. Image semantic segmentation technology offers a more objective and rapid approach to medical image recognition compared to traditional expert diagnosis, reducing the reliance on physician experience and enhancing the standardization of patient diagnosis and treatment.

The U-shaped architectures, featuring encoder-decoder structures, have shown strong performance in segmenting medical representations within images. U-Net [1] leverages skip connections to combine high-level semantics from decoder layers with low-level details from encoder layers. U-Net++ [2] reduces the semantic gap with nested, dense skip connections, while U-Net 3+ [3] captures multi-scale features for comprehensive detail and semantics.

Despite these improvements, the U-Net variants have not fully exploited the semantic segmentation potential of the U-shaped networks. The encoder's feature extraction capabilities and its role in bridging the semantic gap are crucial, mirroring the success of FCNs. This paper focuses on the encoder's unique benefits in the U-shaped networks.

The parameter count in the U-Net series is substantial, with 32 or 64 basic channels, limiting batch size, increasing hardware requirements, and affecting network inference speed. We aim to balance parameter count with segmentation accuracy using minimal parameters.

In this paper, we introduce a lightweight full-encoder U-shaped network (LFE-UNet), with five main contributions: (i) a novel U-shaped network that maximizes encoder feature extraction through full-encoder skip connections; (ii) a lightweight design with 8 basic channels, significantly reducing parameters compared to 32 or 64, to balance network parameters and segmentation accuracy; (iii) an analysis of the impact of Gaussian noise on images and salt and pepper noise on labels, demonstrating our method's robustness across varying data clarity; (iv) an exploration of different loss functions and their combinations, assessing their impact on feature learning and network training; (v) extensive experiments on the BraTS 2018 brain tumor dataset and ISBI LiTS 2017 liver dataset, showing our LFE-UNet's competitiveness despite having fewer parameters.

The remainder of the paper is structured as follows: Section 2 reviews related work. Section 3 details the LFE-UNet architecture and the full-encoder skip connections. Section 4 compares LFE-UNet with other methods on brain tumor and liver datasets, validating its lightweight nature and effectiveness, and discusses the segmentation performance of LFE-UNet with varying basic channels and encoder layers under Vgg16, its robustness to noisy data for liver images and labels, and the impact of different loss functions for brain tumor data. The final section concludes the paper.

## 2 RELATED WORK

To enhance the effective extraction of medical features in imaging, numerous scholars have refined the U-Net architecture [4, 5] and integrated better feature optimization modules for more comprehensive feature extraction.

Kiran *et al*. [6] introduced DenseRes-Unet, integrating dense blocks into U-Net's encoder for segmenting nuclei in histopathology images, focusing on relevant features from previous layers. Yin *et al*. [7] proposed a U-Net variant with squeeze-and-attention (SA) and dense atrous spatial pyramid pooling (Dense ASPP) modules, where SA enhances pixel grouping attention and Dense ASPP captures multi-scale COVID-19 lesion information. Guo *et al*. [8] developed a spatial attention U-Net, incorporating a spatial attention module and structured dropout convolutional blocks to prevent overfitting. Bougourzi *et al*. [9] presented a pyramid dual-decoder attention U-Net with an attention gate, preserving spatial awareness and guiding Covid-19 infection segmen-tation. Maji *et al*. [10] introduced an attention Res-UNet with a guided decoder for brain tumor segmentation, capable of gui-ding each decoder layer's learning process with individual loss functions. Cao *et al*. [11] proposed a U-Net-like pure trans-former, utilizing tokenized image patches for local-global semantic feature learning. Yan *et al*. [12] developed an axial fusion transformer U-Net, combining convolutional layers' detail extraction with transformers' long sequence modeling strength. Chen *et al*. [13] introduced a patches convolution attention-based transformer U-Net, capturing global depen-dencies and feature details through the complementary advan-tages of transformers and CNNs. Khan *et al*. [14] designed a residual U-Net with dilated convolution for probing inter-slice features on a large scale for liver and tumor segmentation.

While these U-Net-based improvements have enhanced segmentation outcomes, they also impose significant computational demands due to extensive network parameters. Additionally, for lightweight medical image segmentation, researchers have proposed a Knowledge Distillation (KD)-based IoMT end-edge-cloud orchestrated architecture [15], and a slimmable transformer to explore intrinsic inductive bias *via* position information [16], among other beyond U-Net-based architectures. Our goal is to propose a lightweight U-Net variant that achieves better or comparable segmentation results with a reduced parameter count.

## 3 METHODS

Fig. (**1**) compares the U-Net series with our proposed LFE-UNet. Distinct from other approaches, our network modifies only the connection scheme, without incorporating additional auxiliary modules, and achieves superior segmentation performance, aligning with the principle of natural simplicity.

The progression of skip connections has advanced from the straightforward skips in the original U-Net, through the nested and dense skips introduced in U-Net++, to the comprehensive full-scale skips featured in U-Net 3+. Our proposed LFE-UNet, however, employs full-encoder skip connections that harness the unique features from all encoder layers. This means that each decoder layer amalgamates features extracted by every encoder layer, fully leveraging the encoder's prowess in feature extraction.

In the U-Net series, the encoder plays a crucial role in capturing both fine-grained details and coarse-grained semantic features across the entire range of the data. Full-encoder skip connections allow the network to preserve rich representations of the input data at different scales. Fine-grained details are essential for identifying small structures and subtle variations in the image, while coarse-grained semantic features provide a broader understanding of the overall context. The introduction of full-encoder skip connections in LFE-UNet aims to capture these features more comprehensively. By integrating features from all encoder layers, the network can better understand both the contextual information and the fine details within the input data.

### 3.1 Expression of Network Parameter Count

The parameter count of our proposed LFE-UNet architecture can be formally expressed as follows: Let *N* denote the total number of encoder layers, and *i* index the down-sampling layers within the encoder. Each encoder layer *X^i^*_En_ extracts features at a smaller scale from the preceding layer, capturing higher semantic information. Each decoder layer *X^i^*_De_ integrates feature maps from all encoder layers at full scale and from the subsequent decoder layer at a larger scale to produce pixel-wise classification results. *X^i^*_De_ is calculated as follows:

**Table:** 

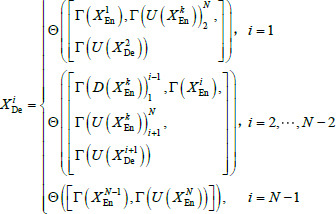	(1)

where Θ(•) realizes a feature aggregation mechanism that encompasses two convolutional operations, followed by batch normalization and a ReLU activation function, Γ(•) represents a single convolution operation, *D*(•) represents a down-sampling operation performed through non-overlapping max-pooling, *U*(•) represents an up-sampling operation achieved *via* bilinear interpolation, and [•] denotes a concatenation operation.

Taking *X*^2^_De_ as an example, as depicted in Fig. (**2**). The feature map of *X*^2^_De_ is constructed by the full-scale encoder layer [*X*^1^_En_, *X*^2^_En_, *X*^3^_En_, *X*^4^_En_, *X*^5^_En_], and the larger-scale decoder layer *X*^3^_De_. This process integrates six resolution levels of feature maps from both the encoder and decoder, effectively merging fine-grained, shallow information with deep, semantic insights.

Mirroring the architecture of U-Net, in LFE-UNet, the number of output channels in the decoder feature map is mirrored from the encoder, meaning that both layers, *X*^*i*^_En_ and *X*^*i*^_De_ possess 2*^i^*^-1^*n* output channels. Consequently, the parameters in the *i*^th^ decoder layer of LFE-UNet, denoted as P*^i^_l_*, can be determined using the following calculation:

**Table:** 

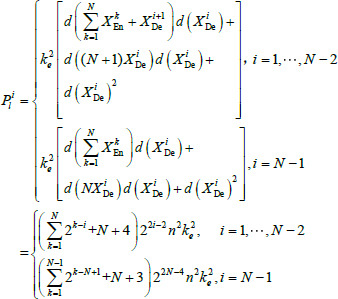	(2)

where *N* denotes the count of encoder layers, *k_e_* signifies the size of the convolution kernel, and *d*(•) represents the number of channels of the notes.

Vgg16 and ResNet34 are selected as the backbone network architectures for LFE-UNet. The parameter count for LFE-UNet integrated with ResNet34, denoted as *P_lv_*, can be calculated as follows:

**Table:** 

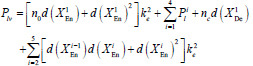	(3)

where *n*_0 _ denotes the number of channels of the input, *n_c_* signifies the number of output classes for the segmentation outcome.

Similarly, the parameter count for LFE-UNet integrated with Vgg16, denoted as *P_lr_*, can be determined as follows:

**Table:** 

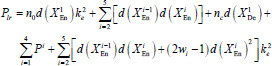	(4)

where *w_i_* denotes the number of Basic blocks for the *i*th layer in ResNet34 and *w* = [*w*_2_ *w*_3_ *w*_4_ *w*_5_] = [3 4 6 3].

### 3.2 Dataset and Evaluation Metric

We selected both binary and multi-class medical imaging datasets to validate the efficacy of our proposed approach.

For the binary classification task, we utilized a liver dataset from the 2017 Liver Tumor Segmentation Benchmark Challenge hosted by the IEEE International Symposium on Biomedical Imaging (ISBI LiTS 2017 Challenge: https://aistudio.baidu.com/aistudio/datasetdetail/ 79729). This dataset comprises 131 contrast-enhanced 3D abdominal CT scans, with 120 and 11 volumes allocated for training and validation, respectively. We extracted the three most prominent slices (512×512) from each volume based on liver size. During training, the input consists of a single slice with 3 channels (*n*_0 _=3), and the output is 2 channels (*n_c_*=2) representing the background and the liver region.

For the multi-class classification task, we chose a brain tumor dataset from the Brain Tumor Segmentation Benchmark (BraTS) 2018 Challenge (https://aistudio.baidu.com/aistudio /datasetdetail/64660). This dataset includes 210 cases of high-grade glioma (HGG) and 75 cases of low-grade glioma (LGG). We extracted all slices (160×160) from each case, excluding slices where the number of pixels classified as enhancing tumor (ET) was less than 20. We used 90% of the slices for training and 10% for validation. The input for training includes 4 slices corresponding to the four modalities of brain MRI—Flair, T1, T1ce (contrast-enhanced T1), and T2—each with 3 channels, resulting in a total of 12 channels (*n*_0 _=12), The output consists of 4 channels (*n_c_*=4), corresponding to the background and three tumor categories: enhancing tumor (ET), peritumoral edema (ED), and non-enhancing tumor (NET). These constitute the nested brain tumor sub-regions: whole tumor (WT), tumor core (TC), and enhancing tumor (ET), as the final segmentation outcome [17]:

**Table:** 

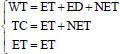	(5)

We employed the Adam optimization algorithm with a learning rate of 1e-4, weight decay of 2e-3, and a batch size adjusted to 5, 2, or 1 based on video memory availability. The training was set for 500 epochs, and the learning rate was decremented to one-tenth of its previous value if there was no improvement on the validation set for 20 consecutive iterations. A NVIDIA GeForce RTX 3090 with 24 GB of memory was utilized for computations.

Drawing upon dice loss, focal loss, and KL divergence loss, the loss function employed is the DFK loss proposed by Deng *et al*. [18]. The dice coefficient was used as the evaluation metric for each segmentation result.

## 4 RESULTS AND DISCUSSION

Q1: Does our proposed network offer any advantages over other methods on the binary-classification dataset?

Q2: Does our proposed network offer any advantages over other methods on the multi-classification dataset?

Q3: How can we achieve a balance between network parameters and segmentation accuracy with minimal parameters?

Q4: Does the proposed network exhibit robustness to datasets containing noisy images?

Q5: Does the proposed network exhibit robustness to datasets with noisy labels?

Q6: What is the impact of different loss functions on the segmentation performance of the proposed network?

### 4.1 Comparison with a Liver Dataset (Q1)

In order to assess the performance of our proposed LFE-UNet, equipped with both Vgg16 and ResNet34 backbones, we conducted a quantitative comparison against other leading-edge methods using the dice coefficient metric on the ISBI LiTS 2017 liver validation dataset. The comparative results are presented in Table **1**.

It should be noted that all results were derived from a single-model test, without the use of any post-processing tools. The input to our segmentation network is directly extracted from the slices, without any filtering or enhancement processing.

As shown in Table **1**, U-Net 3+ with Vgg16 outperforms U-Net++ with Vgg16 by 2.72% using 57.16% of its parameters, and exceeds U-Net with Vgg16 by 3.44% using 68.47% of its parameters. This demonstrates the advantage of full-scale skip connections, with U-Net 3+ having fewer parameters.

In comparison with U-Net 3+, for the Vgg16 backbone, our proposed LFE-UNet with cross-entropy loss surpasses U-Net 3+ by 0.574% using 3.30% of U-Net 3+'s parameters, and LFE-UNet with DFK exceeds it by 0.699%. For the ResNet34 backbone, LFE-UNet with cross-entropy loss outperforms U-Net 3+ with ResNet101, ResNet101 with Hybrid loss, and ResNet101 with Hybrid loss and classification-guided module CGM by 0.893%, 0.473%, and 0.153%, respectively, using 4.43% of U-Net 3+'s parameters. LFE-UNet with DFK surpasses them by 1.375%, 0.955%, and 0.635%, respectively. Additionally, compared to the transformer-enhanced U-Net networks, such as SwinUnet and ResTransUnet, the proposed method demonstrates clear advantages in both *Dice* score and parameter count. Sample results from the proposed LFE-UNet with ResNet34+CE and ResNet34+DFK on the ISBI LiTS 2017 validation dataset are depicted in Fig. (**3**).

In Fig. (**3**), it is visually evident that, compared to the ground truth labels, the proposed LFE-UNet based on ResNet34 performs well in segmenting and identifying the liver region under both cross-entropy loss and DFK loss. The cross-entropy loss is particularly effective in identifying the central depression, while the DFK loss better compensates for the lack of detail in the external regions, especially for the identification of the upper right tip.

Consequently, for the binary liver dataset, our lightweight LFE-UNet network demonstrates a slightly stronger segmenta-tion capability than U-Net 3+, validating the effectiveness of our network structure and the benefits of leveraging features from all encoder layers for segmentation performance.

### 4.2 Comparison with a Brain Tumor Dataset (Q2)

We acquired dice coefficients for the three nested brain tumor sub-regions on the BraTS 2018 validation dataset by training the LFE-UNet with Vgg16 and ResNet34. Furthermore, to validate the effectiveness of our proposed network, we compared our results with other state-of-the-art methods, as shown in Table **2**. Fig. (**4**) displays some results obtained using the LFE-UNet with ResNet34 and DFK on the validation dataset, and its performance in identifying the outer contours in the gray areas and the internal black regions is very similar to that of the label map.

In Table **2**, our LFE-UNet, augmented with ResNet34 and DFK, outperforms other state-of-the-art methods, achieving superior average performance on the three nested brain tumor sub-regions. It surpasses Myronenko's ensemble method by 0.840% and No New-Net by 1.353%. Specifically, it excels in WT and TC sub-regions, with dice coefficients of 93.759% and 87.301%, respectively, which are 2.499% higher than No New-Net for WT and 0.621% higher than Myronenko's ensemble method for TC. Notably, the LFE-UNet with ResNet34 and CE ranks second in average, WT, and TC, while the LFE-UNet with ResNet34 and DFK ranks second in ET. Moreover, with only 1.93M parameters, which is just 59.02% of the smallest network with 3.27M parameters, our LFE-UNet demonstrates equal or better performance with approximately half the parameters of existing networks, proving the effectiveness of the encoder in enhancing performance.

Furthermore, the LFE-UNet with ResNet34 outperforms the version with Vgg16, and with DFK, it improves average, WT, TC, and ET performances by 2.234%, 1.133%, 2.640%, and 2.929%, respectively. With CE, it improves these metrics by 3.132%, 1.413%, 5.516%, and 2.469%, respectively. These results highlight the general effectiveness and advantages of residual networks.

In conclusion, the LFE-UNet with ResNet34 and DFK demonstrates equal or superior recognition capabilities for medical representations on both binary liver and multi-class brain tumor datasets compared to existing networks. With less than 5% of the parameters of U-Net series, it confirms the lightweight nature of our proposed method and the unique advantages of full encoder features in U-shaped networks.

### 4.3 Network Parameters (Q3)

To strike a balance between network parameters and segmentation accuracy, we focus on the number of basic channels *n* and the number of encoder layers *N*, both of which are directly proportional to the network parameters.

To determine the network parameters, using Equation (3) as a reference, the parameter count *P^N^_lv_* for the LFE-UNet network with a Vgg16 backbone and an *N*-layer encoder is derived as follows:

**Table:** 

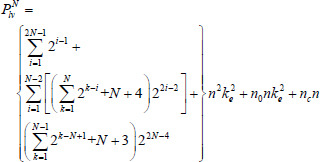	(6)

Considering the number of basic channels *n* and the number of encoder layers *N*, the optimal dice coefficients and the corresponding training times for the LFE-UNet with a Vgg16 backbone on the ISBI LiTS 2017 validation dataset are presented in Table **3**.

In analyzing Table **3** with respect to parameter count and training time, it is observed that when the number of encoder layers *N* is held constant, an increase in the number of basic channels *n*, leads to an approximate fourfold increase in network parameters, and the total training time also increases, with the rate of increase being directly proportional to the size of *n*. Conversely, when the number of basic channels *n* is held constant, an increase in the number of encoder layers *N* results in an approximate 4.5±0.22-fold increase in network parame-ters, with a corresponding increase in total training time.

Regarding segmentation performance, when the number of encoder layers *N* is constant, a slight improvement in segmentation effect is noted with an increase in the number of basic channels *n*, but this improvement must be weighed against the training time. For instance, when *N* is set to 5, the training time for *n* = 8 is only 42% of that for *n* = 32, with a minimal decrease in the segmentation effect of just 0.284%. Moreover, when *n* = 8, the parameter count is only 25% of that for *n* = 16, yet the segmentation effect improves by 0.188%. Furthermore, increasing *N* from 3 to 5 significantly enhances segmentation performance, but further increasing *N* to 6 yields only a modest improvement. When *n* is set to 64, the segmentation effect is markedly poor, falling below 60%, likely due to memory constraints caused by the large parameter count. At this point, the batch size is limited to 1, which means the network can only approximate the true value of a single slice at a time and fails to capture the horizontal information shared between different slices. While this may lead to higher training effects, the validation effect is considerably lower.

In summary, the analysis reveals that when the number of encoder layers *N* is set to 5 and the number of basic channels *n* is set to 8, the LFE-UNet achieves a higher *Dice* value with a notably short training time of 3.25 hours. This combination significantly outperforms other parameter configurations, effectively reconciling the trade-off between network parameters and segmentation accuracy.

To provide a more intuitive comparison of the training process for each parameter combination, the loss value change curves on the validation dataset corresponding to different numbers of basic channels *n* for each encoder layer count *N* were plotted, as depicted in Fig. (**5**).

Fig. (**5a** and **b**) show that the loss values for *n* = 8 and *n* = 16 converge to nearly the same level, whereas the convergence for *n* = 32 and *n* = 64 is lower. Fig. (**5c** and **d**) indicate that the number of basic channels *n* has minimal impact on the convergence level of the loss value. Observing the differences in *N* across Fig. (**5**), it is evident that the convergence level of the loss value significantly decreases step by step when *N* takes values of 3, 4, and 5, and remains relatively consistent between *N* = 5 and *N* = 6.

Furthermore, for the cases where *N* is set to 3, 4, or 5, it is observed that with *n* = 8, both the learning and the rate of decline are swifter compared to when *n* = 16. Conversely, with *n* = 64, the loss value not only starts at the lowest point but also declines most rapidly. When *N* = 6, the decline in loss is initially faster for *n* = 16 than for *n* = 8, though they eventually align later on. However, with *n* = 64, both the initial loss value and the final convergence level are the highest, which substantiates the poor validation outcome mentioned earlier, attributed to the substantial parameter count and the constraints of video memory. Fig. (**6**) displays segmentation outcomes from the LFE-UNet with various parameter configurations on the ISBI LiTS 2017 validation dataset, illustrating that the configuration with *N* = 5 and *n* = 8 yields performance that is at least equivalent, if not superior.

Hence, when the number of encoder layers *N* is set to 5 and the number of basic channels *n* is set to 8, the proposed LFE-UNet demonstrates a lower convergence level compared to most other configurations. It maintains a respectable convergence speed and benefits from a reduced parameter count, which is not constrained by memory limitations. These findings affirm that this particular parameter combination offers superior cost-performance within the context of a lightweight network architecture.

### 4.4 The Impact of Images with Varying Clarity (Q4)

With *N* set to 5 and *n* set to 8, we explored the impact of to-be-learned images with varying clarity on the segmentation performance of the LFE-UNet with a Vgg16 backbone network.

Recognizing that quantum noise in CT images stems from a signal-dependent Poisson-distributed noise source, and that this Poisson noise can be approximated by a Gaussian distribution when the quantum count is large [33]. We introduced Gaussian noise into the slices of contrast-enhanced 3D abdominal CT scans, which serve as the images. Different variances *v_r_* were set to create a series of new liver datasets with varying degrees of image clarity.

The images are essentially noise-free for *v_r_* < 0.01, and completely obscured by noise for *v_r_* = 1 at the extreme values. The variances of the Gaussian noise tested were 0.01, 0.02, 0.04, 0.06, 0.08, 0.1, 0.2, 0.4, 0.6, 0.8, as illustrated in Fig. (**7**).

In Fig. (**7**), as the variance of Gaussian noise increases, image clarity correspondingly decreases. When *v_r_* > 0.08, details in the images become indistinguishable to the naked eye.

For these new liver datasets with varying clarity, the optimal dice coefficients achieved by the LFE-UNet with Vgg16 on the ISBI LiTS 2017 validation dataset are detailed in Table **4**.

In Table **4**, as the variance of Gaussian noise in the images increases, the learning capability of the LFE-UNet diminishes, consequently degrading the segmentation performance on the liver validation dataset.

Compared to images without Gaussian noise, the segmentation effect under images with *v_r_* = 0.01 decreases by only 0.800%, well within a 1% margin. Under images with *v_r_* = 0.02,0.04,0.06, the segmentation effect decreases by 1.895%, 2.968%, and 2.893%, respectively, all within a 3% margin. Under images with *v_r_* = 0.08,0.1, the segmentation effect decreases by 3.426% and 4.233%, respectively, both within a 5% margin. It is evident that slight noise in the images has a negligible impact on the segmentation performance of the proposed LFE-UNet, and the effect of minor noise is within an acceptable range.

Under images with *v_r_* ≤ 0.1, the LFE-UNet achieves segmentation effects on liver validation datasets that all exceed 90%. For images with 0.1 < *v_r_* < 0.8, the segmentation effects all exceed 80%, and for images with *v_r_* = 0.8, the performance is close to 80%. Despite this, for the human eye, the details in images with *v_r_* > 0.08 are considerably blurred, making it challenging to discern the specific area where the liver is located with the naked eye. These observations demonstrate that the LFE-UNet does not demand high precision in the images and possesses a certain degree of robustness against noise.

To provide a more intuitive comparison of the training process for to-be-learned images with different clarity, the change curves of the loss value on the validation dataset were plotted, as shown in Fig. (**8**).

In Fig. (**8**), the learning speeds of the LFE-UNet are relatively consistent across different variances of Gaussian noise in the to-be-learned images. However, as the variance of the Gaussian noise increases, the convergent loss value on the validation dataset also increases, corresponding to a weakening of the segmentation effect. Notably, under *v_r_* ≤ 0.1, the convergent loss value on the validation dataset remains almost the same (within 0.03 difference), indicating that the segmentation effect is similar to that without noise.

Some segmentation results obtained on the ISBI LiTS 2017 validation dataset are displayed in Fig. (**9**).

In Fig. (**9**), as the variance of Gaussian noise in the to-be-learned images increases, the learning of liver region details in the segmentation result gradually diminishes, but the liver's contour remains relatively intact.

Therefore, considering both the network training process and the actual liver segmentation outcomes, the LFE-UNet is effective in recognizing images with low definition and exhibits a certain level of robustness.

### 4.5 The Impact of Labels with Varying Clarity (Q5)

Owing to the inherent complexity of medical image structures and the high difficulty and labor intensity involved in segmenting the target areas, it is inevitable that noise will be present in labels [34].

Building on a configuration with *N* = 5 and *n* = 8, we examined the impact of labels with varying clarity on the segmentation efficacy of the LFE-UNet with a Vgg16 backbone.

Given the binary nature of the labels, salt and pepper noise was introduced into the liver dataset labels, with varying degrees of noise intensity represented by different variances *v_s_* to create a series of new liver datasets with labels of differing clarity levels.

Mirroring the variances of Gaussian noise set for the images, the variances of salt and pepper noise were set at 0.01, 0.02, 0.04, 0.06, 0.08, 0.1, 0.2, 0.4, 0.6, 0.8, as depicted in Fig. (**10**).

For these new liver datasets with varying label clarity, the optimal dice coefficients achieved by the LFE-UNet with Vgg16 on the ISBI LiTS 2017 validation dataset post-training are detailed in Table **5**.

In Table **5**, as the variance of salt and pepper noise in the labels increases, the learning capability of the LFE-UNet exhibits slight fluctuations but remains stable at around 95%. This indicates that the network's segmentation performance on the liver validation dataset is not significantly affected by the variance of salt and pepper noise, maintaining a dynamic equilibrium.

The segmentation effects under labels with varying degrees of salt and pepper noise (*v_s_* = 0.01,0.02,0.04,0.06,0.08,0.1,0.2, 0.4,0.6,0.8) compared to noise-free labels show differences of -0.055%, -1.105%, -0.636%, +0.085%, -0.166%, -0.004%, -0.681%, -0.482%, +0.401%, and -0.185%, respectively, with all variations falling within the ±1.5% range. These minor fluctuations are attributed to the stochastic nature of the training process. This indicates that the LFE-UNet does not demand highly accurate labels and exhibits strong robustness against label noise.

To provide a more intuitive comparison of the training process under noisy labels, the change curves of the loss value on the validation dataset were plotted, as shown in Fig. (**11**).

In Fig. (**11**), as the variance of salt and pepper noise increases, the convergent loss value on the validation dataset also increases, but the segmentation effects remain in dynamic balance. The widening gap between noisy labels and true labels in the training dataset due to increased noise variance leads to a continuous increase in the loss value obtained through training. Since the discrepancy between predictions and labels in the training dataset is progressively narrowing during the learning process, the corresponding loss value on the validation dataset may not be too low, even if the difference between predictions and true labels on the validation dataset is minimal. Thus, the correlation between the loss value and segmentation effect is not significant in this context, and direct comparability is limited.

Some segmentation results obtained on the ISBI LiTS 2017 validation dataset are displayed in Fig. (**12**).

Fig. (**12**) demonstrates that the LFE-UNet, when trained under labels with varying levels of salt and pepper noise, yields segmentation results that are largely consistent. The outer contour and internal detailed features of the liver region are clearly identifiable, even in the presence of a significant amount of visibly interfering noise. This intuitively shows that there is essentially no impact on the actual segmentation performance of the LFE-UNet due to noisy labels.

Consequently, the LFE-UNet is capable of effectively managing the relationship between noise and the target area, exhibiting a certain degree of robustness against noise in both the images and the labels.

### 4.6 Discussion of the Loss Function (Q6)

In this study, we have selected 9 loss functions to evaluate their impact on the performance of the LFE-UNet. These include Cross-Entropy Loss (CE), L2 Loss, Smooth F1 Loss (SF), Binary Cross-Entropy Loss (BCE), Binary Cross-Entropy with Logits Loss (BCL), Dice Loss (DL), Tversky Loss (TL), Focal Loss (FL), and Kullback-Leibler Divergence Loss (KL). These functions are expressed as follows [35]:

**Table:** 

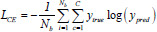	(7)

**Table:** 

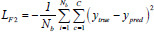	(8)

**Table:** 

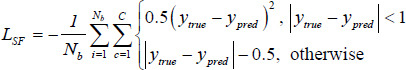	(9)

**Table:** 

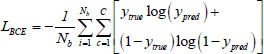	(10)

**Table:** 

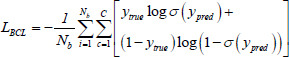	(11)

**Table:** 

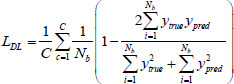	(12)

**Table:** 

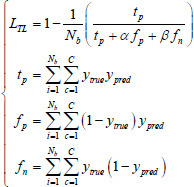	(13)

**Table:** 

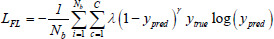	(14)

**Table:** 

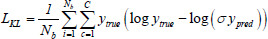	(15)

where *y*_true_ denotes the actual label for the segmentation task, *y*_pred_ signifies the predicted label resulting from the segmentation, *N_b_* indicates the batch size utilized during training, and *C* represents the total number of classes for segmentation, σ refers to the sigmoid activation function, *α* and *β* are hyperparameters associated with the TL, assigned values of 0.3 and 0.7, respectively, *λ* is the balance factor for the FL, set to 0.25, and *γ* is the exponential factor of the FL, with a value of 2.

The dice coefficients resulting from the segmentation performed by the LFE-UNet, employing both Vgg16 and ResNet34 backbones with various loss functions on the BraTS 2018 validation dataset, are presented in Tables **6** and **7**, respectively.

In Table **6**, the combination of FL and KL achieves the best average performance at 84.901%, closely followed by the combination of DL and KL at 84.841%, with a minimal difference of only 0.060%. Among individual loss functions, FL outperforms with an average of 84.436%, slightly ahead of DL at 84.057%, with a difference of 0.379%. For individual sub-regions, the combination of CE and KL excels on WT with 93.156%, nearly matching the combination of FL and KL at 93.142%, differing by a mere 0.014%. The combination of CE and FL leads to TC with 84.289%, followed by FL alone at 83.961%, with a difference of 0.328%. DL dominates on ET, achieving 78.985%, followed by the combination of DL and KL at 78.114%, with a difference of 0.871%. Thus, KL, FL, and DL each contribute significantly to the LFE-UNet with Vgg16 in segmenting the three nested brain tumor sub-regions, as reflected in both individual and average performance.

In Table **7**, the top three average performances are achieved by the combinations of FL and DL at 87.476%, FL and KL at 87.445%, and DL and KL at 87.430%, with differences of only 0.031% and 0.015%. FL leads among individual loss functions with an average of 87.384%, slightly ahead of dice loss at 87.348%, with a difference of 0.036%. For individual sub-regions, the combinations of FL and DL, FL and KL, and DL and KL secure the top three performances on WT at 93.757%, 93.742%, and 93.730%, respectively, with differences of only 0.015% and 0.012%, and on ET at 81.438%, 81.428%, and 81.416%, respectively, with differences of only 0.010% and 0.012%. The combinations of CE and DL, FL, and DL achieve the top three performances on TC at 87.481%, 87.415%, and 87.255%, respectively, with differences of only 0.066% and 0.160%. Thus, KL, FL, and DL also play crucial roles for the LFE-UNet with ResNet34 in the semantic segmentation of brain tumor sub-regions.

In summary, the selection of the DFK loss function proposed by Deng *et al*. to enhance the semantic segmentation capabilities of the LFE-UNet in brain tumor sub-regions is well-founded.

## CONCLUSION

In this study, we introduced a novel LFE-UNet featuring full-encoder skip connections to fully leverage the feature extraction capabilities of a comprehensive encoder. Coupled with a lightweight parameter design and the loss function combination DFK, our model outperforms or is on par with existing networks on both binary liver segmentation datasets and multi-class brain tumor segmentation datasets. This underscores the unique strengths of the encoder in feature extraction within U-shaped network architectures. We explored various combinations of basic channel numbers *n* and encoder layer counts *N*, demonstrating that a configuration with *N* = 5 and *n* = 8 strikes an optimal balance between network parameters and segmentation accuracy, achieving a lightweight model. We also discussed the impact of varying clarities in images and labels, confirming the robustness of the LFE-UNet. With both Vgg16 and ResNet34 as backbone networks, we evaluated multiple loss functions and their combinations to assess their influence on our method. The results justify the adoption of the DFK loss function combination for our proposed LFE-UNet, which capitalizes on the strengths of exponential and logarithmic functions. The current study mainly focuses on liver and brain tumor segmentation datasets, with promising results. However, the generalizability of LFE-UNet to other medical imaging tasks and datasets needs further validation. Future work should include extensive experiments on a wider range of medical imaging datasets from various anatomical regions (*e.g*., cardiac, musculoskeletal) and modalities (*e.g*., MRI, ultrasound) to better assess the model's robustness and adaptability in different clinical settings.

## Figures and Tables

**Fig. (1):**
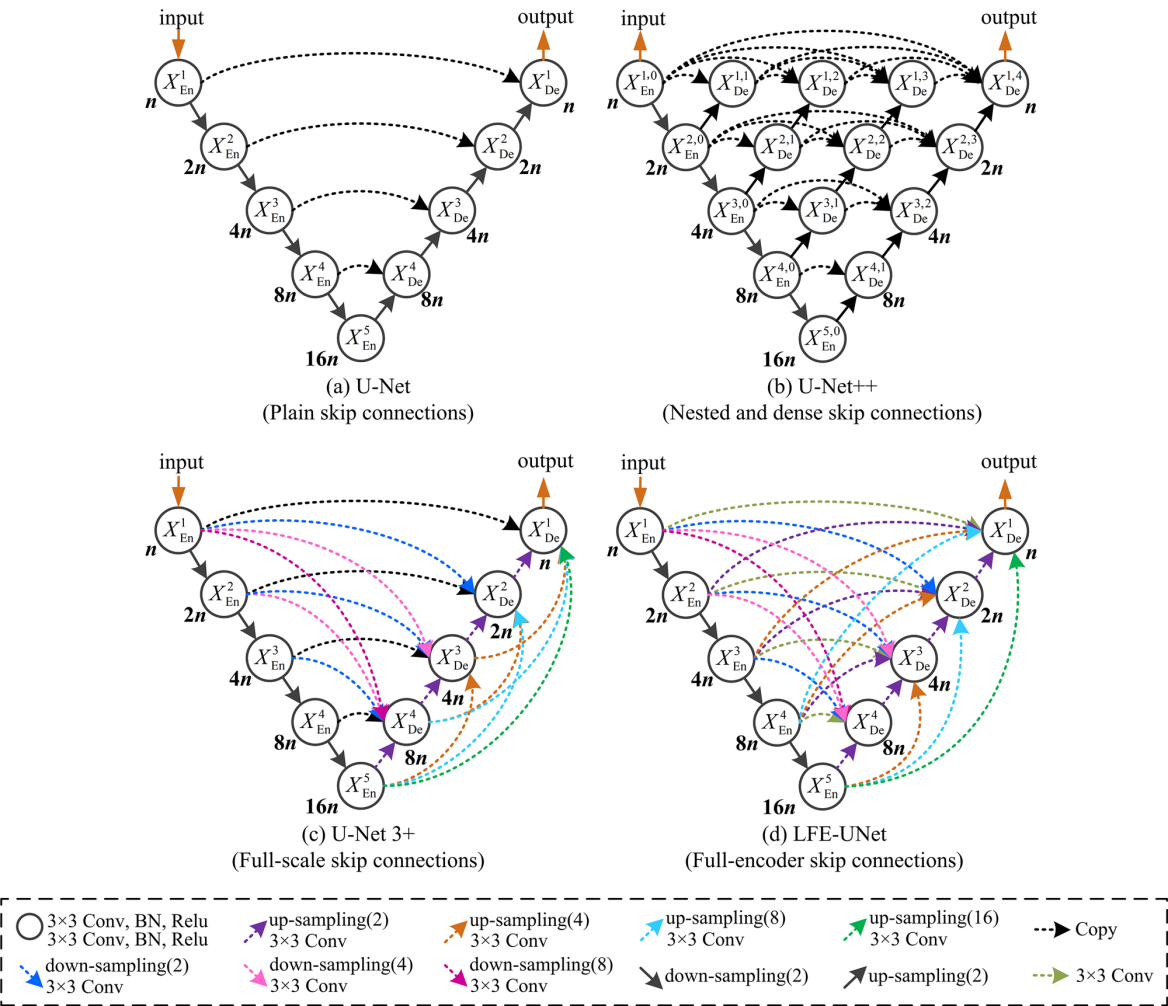
Comparative diagram of U-Net [1] (**a**), U-Net++ [2] (**b**), U-Net 3+ [3] (**c**), and the proposed LFE-UNet (**d**). The number of output channels for each node is indicated beneath the corresponding circle.

**Fig. (2):**
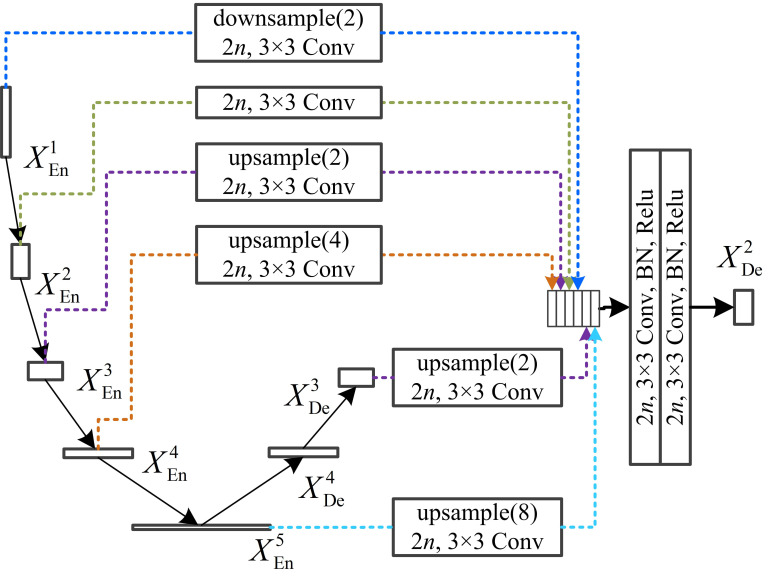
Schematic representation of the construction process for the aggregated feature map at the second decoder layer, utilizing dotted lines to denote the origins of the feature maps.

**Fig. (3):**
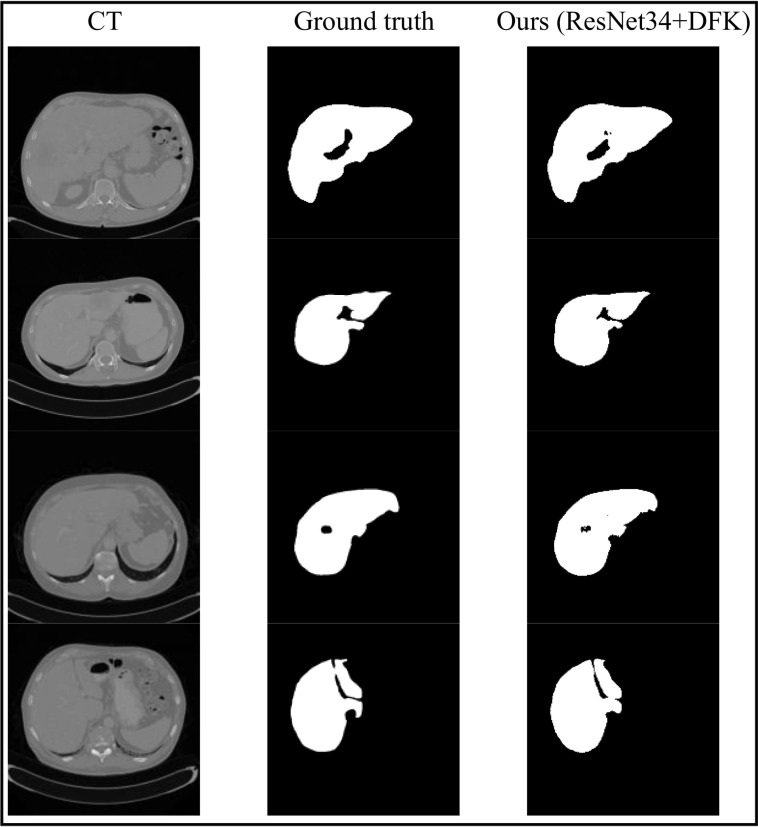
Segmentation results on the ISBI LiTS 2017 validation dataset using LFE-UNet with ResNet34+CE and ResNet34+DFK.

**Fig. (4):**
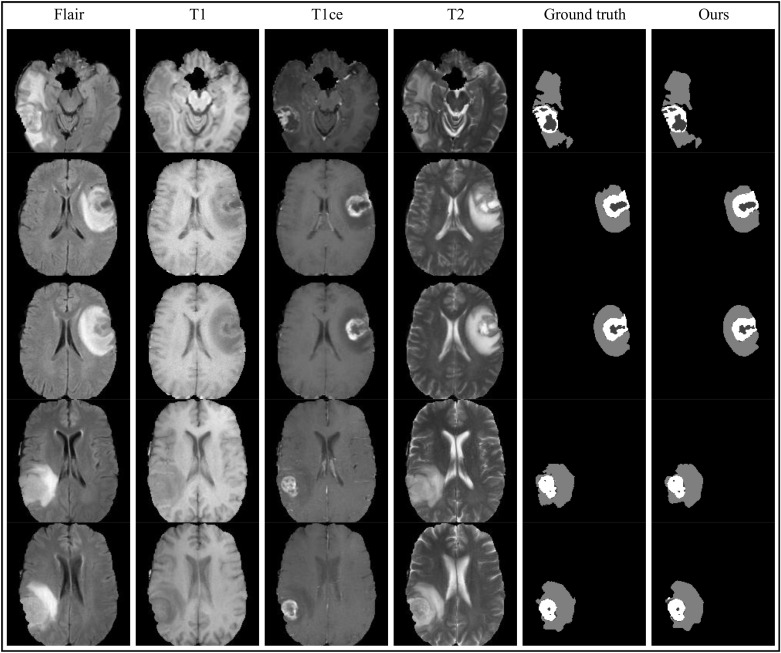
Segmentation results on the BraTS 2018 validation dataset achieved by the LFE-UNet with ResNet34 and DFK.

**Fig. (5):**
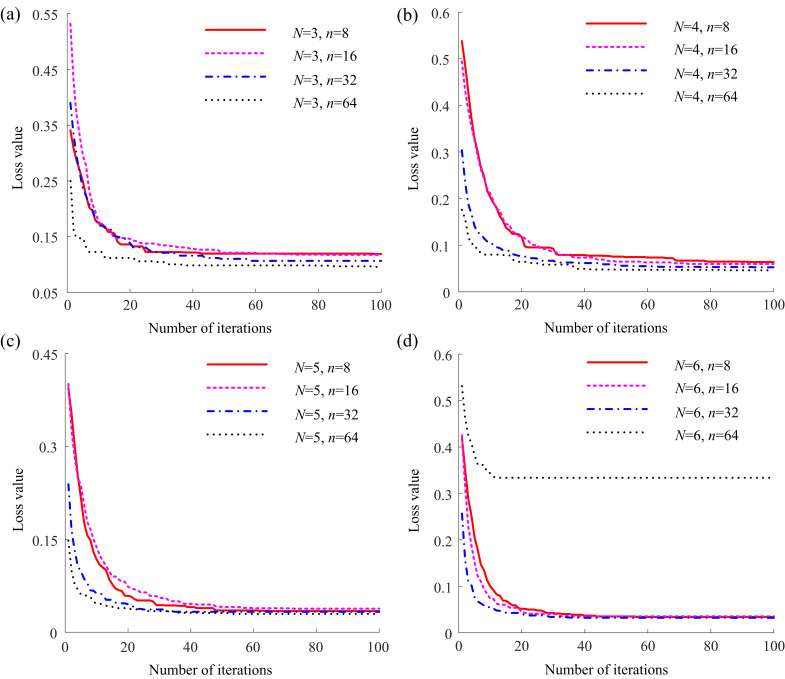
Loss curves on the ISBI LiTS 2017 validation dataset for the our network with *N* = 3 (**a**), *N* = 4 (**b**), *N* = 5 (**c**), and *N* = 6 (**d**).

**Fig. (6):**
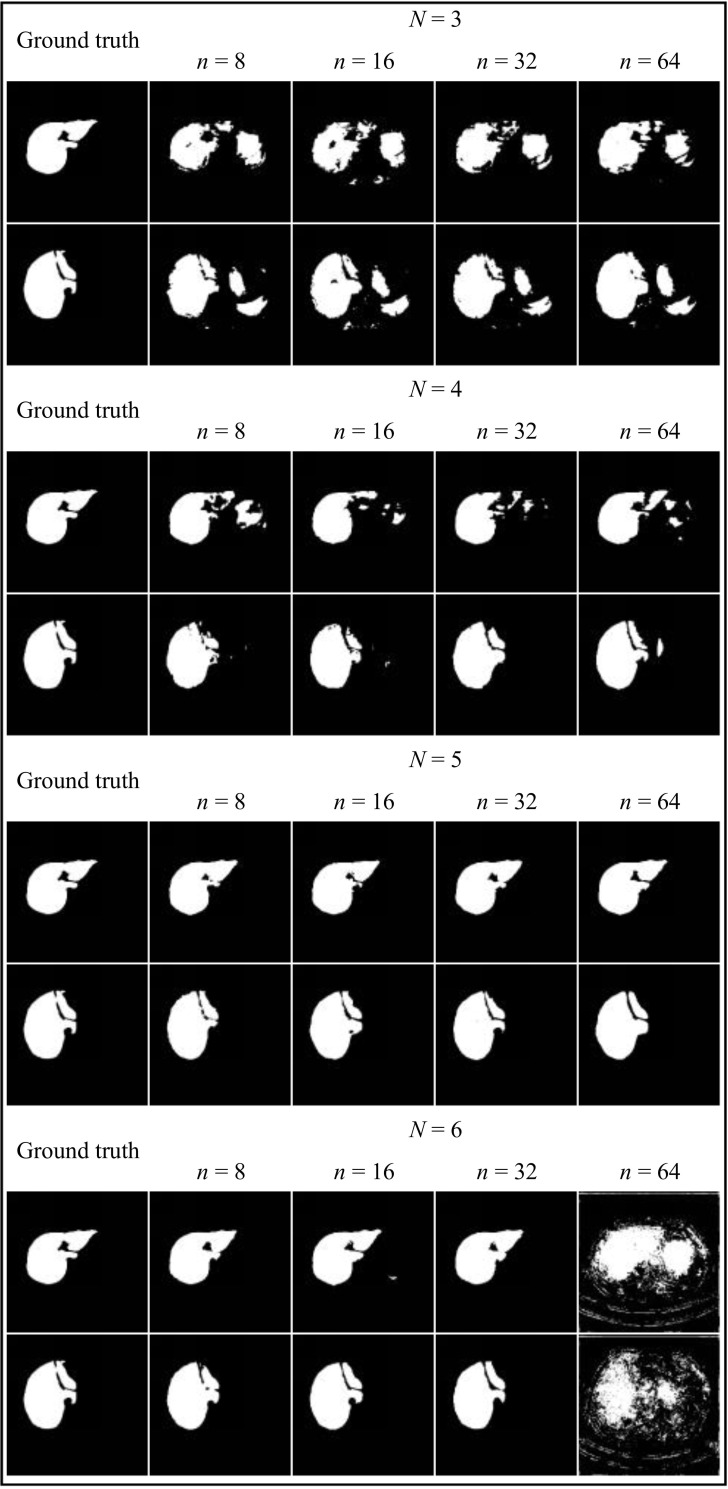
Segmentation results for the LFE-UNet with Vgg16 under different parameter combinations on the liver validation dataset.

**Fig. (7):**
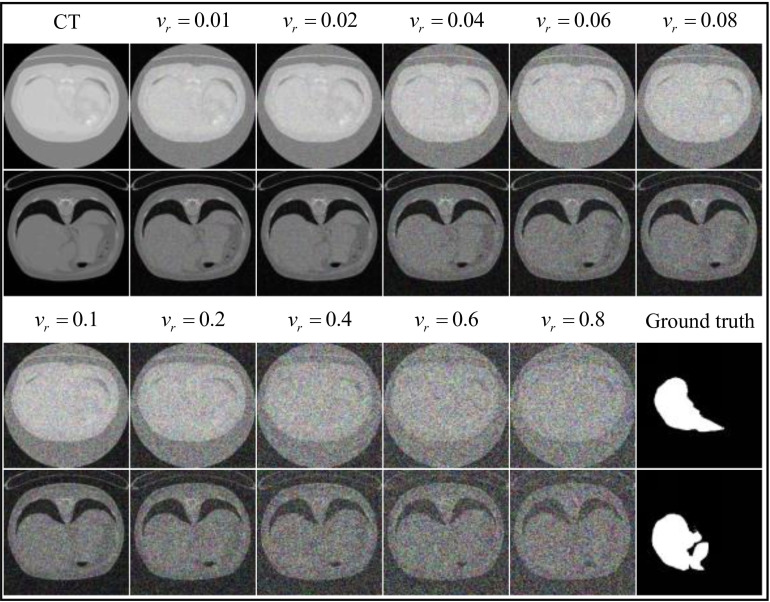
The gaussian noise operation for the to-be-learned images on the liver dataset.

**Fig. (8):**
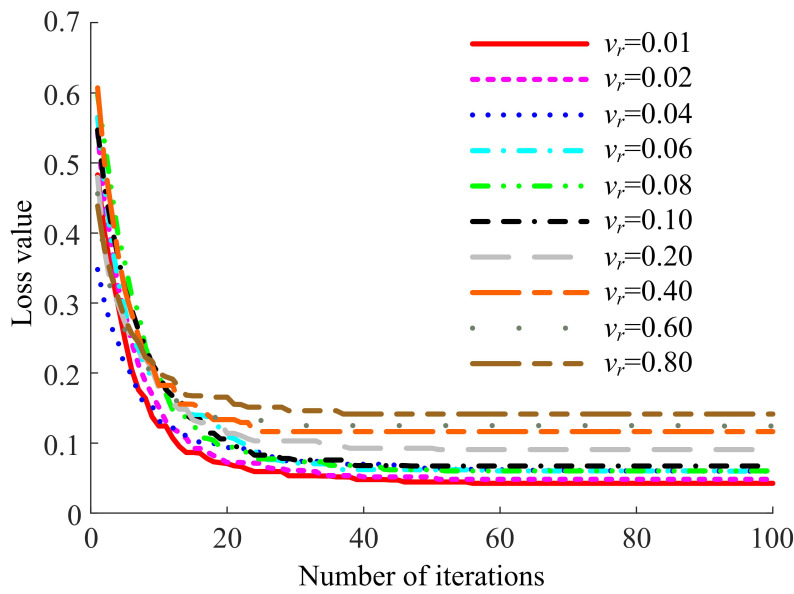
Loss curves on the ISBI LiTS 2017 validation dataset for the LFE-UNet with Vgg16 under images with varying Gaussian noise.

**Fig. (9):**
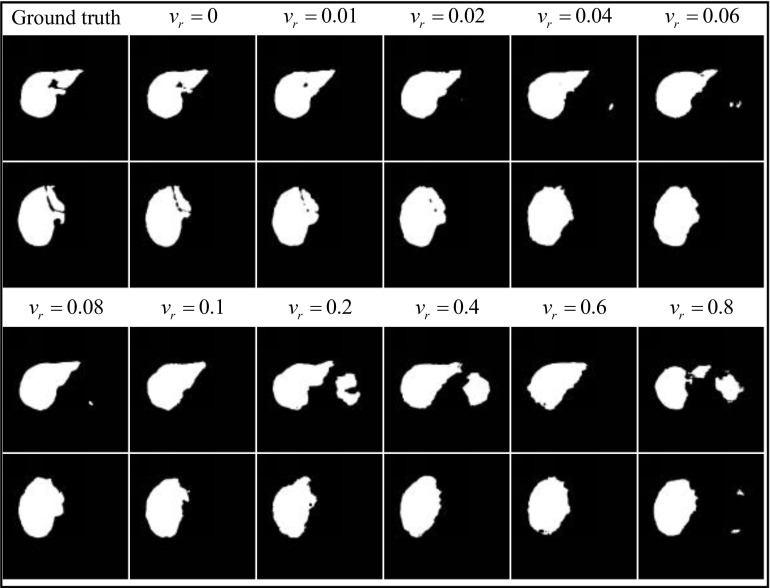
Segmentation results for the LFE-UNet with Vgg16 on the liver validation dataset under images with different Gaussian noise.

**Fig. (10):**
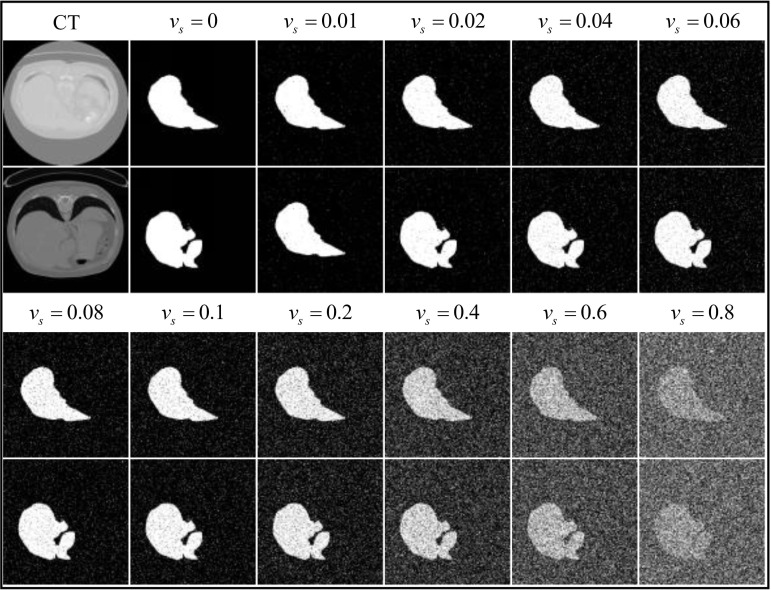
Illustration of salt and pepper noise operation on the liver dataset labels.

**Fig. (11):**
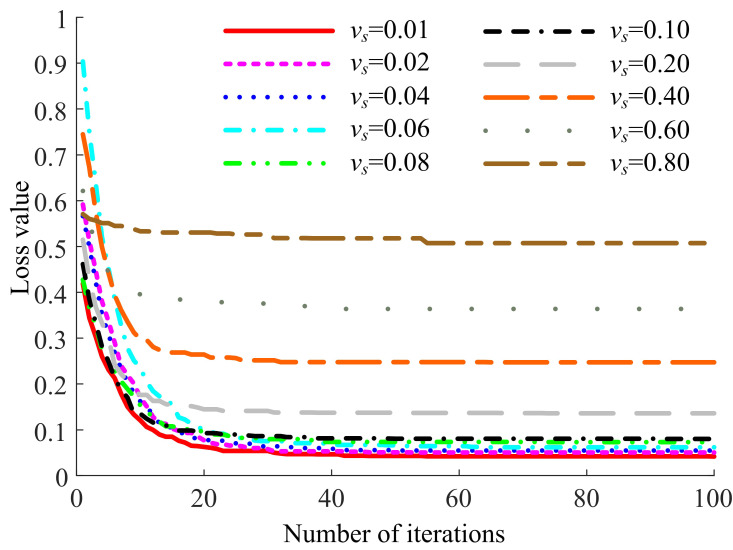
Loss curves on the ISBI LiTS 2017 validation dataset for the LFE-UNet with Vgg16 under labels with varying salt and pepper noise levels.

**Fig. (12):**
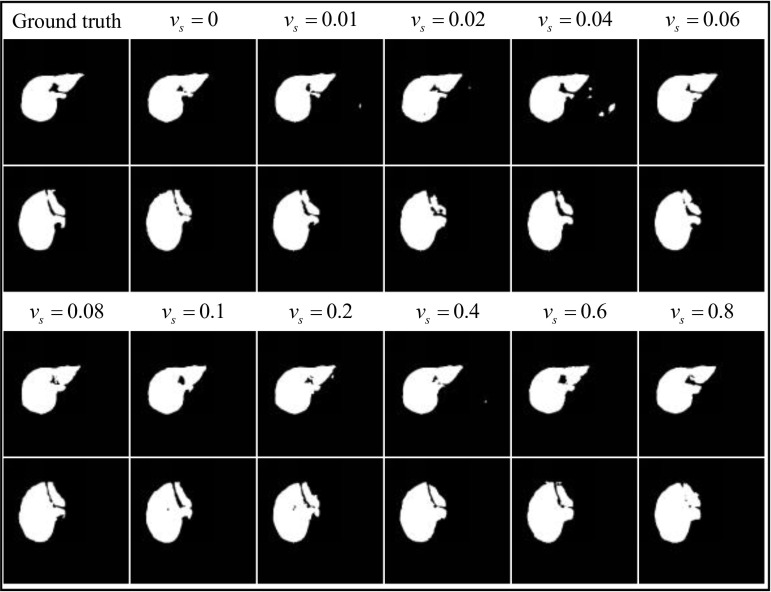
The segmentation result for the LFE-UNet with Vgg16 on liver validation dataset under the labels with different salt and pepper noise.

**Table 1:** Comparison of our methods with state-of-the-art approaches on the ISBI LiTS 2017 validation dataset, with the top two results highlighted in bold and underlined, respectively.

Method	Dice	Params (M)
PSPNet [19]	0.92420	-
DeepLabV2 [20]	0.90210	-
DeepLabV3 [21]	0.92170	-
DeepLabV3+ [22]	0.91860	-
U-Net (Vgg16) [1]	0.92060	39.39
U-Net (ResNet101) [1]	0.93870	55.90
Attention U-Net [23]	0.93410	39.74
U-Net++ (Vgg16) [2]	0.92780	47.18
U-Net++ (ResNet101) [2]	0.94750	63.76
U-Net 3+ (Vgg16) [3]	0.95500	26.97
U-Net 3+ (ResNet101) [3]	0.96010	43.55
U-Net 3+ (ResNet101+Hybrid loss) [3]	0.96430	43.55
U-Net 3+ (ResNet101+Hybrid loss+CGM) [3]	0.96750	43.55
SwinUnet [11]	0.94710	43.44
ResTransUnet [24]	0.95350	53.75
LFE-UNet (Vgg16+CE)	0.96074	**0.89**
LFE-UNet (Vgg16+DFK)	0.96199	**0.89**
LFE-UNet (ResNet34+CE)	0.96903	1.93
LFE-UNet **(ResNet34+DFK)**	**0.97385**	1.93

**Note**: * CGM is the classification-guided module [3].

**Table 2:** Comparative analysis of our methods versus other state-of-the-art methods on the BraTS 2018 validation dataset.

Method	Mean	WT	TC	ET	Params (M)
McKinley *et al*. [25]	0.84867	0.90300	0.84700	0.79600	-
Zhou *et al*. [26]	0.85880	0.90780	0.85750	0.81111	13.81
Kermi *et al*. [27]	0.81867	0.86800	0.80500	0.78300	10.16
Albiol *et al*. [28] (Vgg16)	0.79433	0.87200	0.76000	0.75100	3.27
Albiol *et al*. [28] (Ensemble)	0.81033	0.88100	0.77700	0.77300	6.75
Feng *et al*. [29]	0.84577	0.90940	0.83620	0.79170	-
Ahmad *et al*. [30]	0.85401	0.90715	0.84115	0.81374	3.70
Myronenko [31]. (Single)	0.85943	0.90420	0.85960	0.81450	-
Myronenko [31]. (Ensemble)	0.86670	0.91000	0.86680	**0.82330**	-
No New-Net [32]	0.86157	0.91260	0.86340	0.80870	-
LFE-UNet (Vgg16+CE)	0.83553	0.92085	0.81523	0.77050	**0.89**
LFE-UNet (Vgg16+DFK)	0.85276	0.92626	0.84661	0.78540	**0.89**
LFE-UNet (ResNet34+CE)	0.86685	0.93498	0.87039	0.79519	1.93
LFE-UNet (ResNet34+DFK)	**0.87510**	**0.93759**	**0.87301**	0.81469	1.93

**Table 3:** Dice coefficients for the segmentation results of LFE-UNet with vgg16 backbone on the ISBI LiTS 2017 validation dataset.

*N*	*n*	*P^N^_lv_* (M)	*Dice*	Time (h)
3	8	0.043	0.81776	1.80
	16	0.173	0.81719	1.72
	32	0.692	0.83672	2.24
	64	2.767	0.85321	3.51
4	8	0.203	0.91097	2.17
	16	0.811	0.91300	2.36
	32	3.245	0.92700	3.57
	64	12.978	0.93710	6.64
5	8	0.892	0.95440	3.25
	16	3.569	0.95252	4.35
	32	14.277	0.95724	7.65
	64	57.104	0.96028	15.78
6	8	3.826	0.95795	7.67
	16	15.304	0.95413	12.92
	32	61.214	0.95902	27.65
	64	244.853	0.59141	53.87

**Table 4:** Dice coefficients for the LFE-UNet with Vgg16 on the ISBI LiTS 2017 validation dataset under different levels of Gaussian noise.

*v_r_*	*v_r_* = 0.01	*v_r_* = 0.02	*v_r_* = 0.04	*v_r_* = 0.06	*v_r_* = 0.08
*Dice*	0.94640	0.93545	0.92472	0.92547	0.92014
*v_r_*	*v_r_* = 0.1	*v_r_* = 0.2	*v_r_* = 0.4	*v_r_* = 0.6	*v_r_* = 0.8
*Dice*	0.91207	0.87413	0.83375	0.82598	0.79959

**Table 5:** Dice coefficients for the LFE-UNet with Vgg16 on the ISBI LiTS 2017 validation dataset under labels with different salt and pepper noise intensities.

*v* *_s_*	*v* *_s_*	*v* *_s_* =0.02	*v* *_s_* =0.04	*v* *_s_* =0.06	*v* *_s_* =0.08
*Dice*	0.95385	0.94335	0.94804	0.95525	0.95274
*v* *_s_*	*v* *_s_* =0.1	*v* *_s_* =0.2	*v* *_s_* =0.4	*v* *_s_* =0.6	*v* *_s_* =0.8
*Dice*	0.95436	0.94759	0.94958	0.95841	0.95255

**Table 6:** Dice coefficients for the segmentation results obtained by the LFE-UNet with a Vgg16 backbone using different loss functions on the BraTS 2018 validation dataset. The top two results are highlighted in bold and underlined, respectively.

Loss function									*Dice*			
CE	F2	SL	BCE	BCL	DL	TL	FL	KL	Mean	WT	TC	ET
√									0.83553	0.92085	0.81523	0.77050
	√								0.80057	0.89885	0.78095	0.72192
		√							0.81520	0.91506	0.80237	0.72815
			√						0.83437	0.91294	0.82617	0.76401
				√					0.83732	0.91644	0.82968	0.76583
					√				0.84057	0.91386	0.81799	**0.78985**
						√			0.76883	0.87630	0.71996	0.71022
							√		0.84436	0.92388	0.83961	0.76960
								√	0.82611	0.91429	0.79945	0.76458
√			√						0.83766	0.91599	0.82981	0.76717
√				√					0.83739	0.91788	0.81917	0.77513
√					√				0.84134	0.92134	0.83032	0.77236
√							√		0.84470	0.92548	**0.84289**	0.76574
√								√	0.84327	**0.93156**	0.82415	0.77410
					√		√		0.84443	0.92524	0.83557	0.77249
							√	√	**0.84901**	0.93142	0.83706	0.77855
					√			√	0.84841	0.92540	0.83870	0.78114

**Table 7:** Dice coefficients for the segmentation results obtained by the LFE-UNet with a ResNet34 backbone using different loss functions on the BraTS 2018 validation dataset. The top two results are highlighted in bold and underlined, respectively.

Loss function									Dice			
CE	F2	SL	BCE	BCL	DL	TL	FL	KL	Mean	WT	TC	ET
√									0.86685	0.93498	0.87039	0.79519
	√								0.85494	0.92976	0.85458	0.78048
		√							0.83437	0.91949	0.84256	0.74104
			√						0.85933	0.93375	0.85007	0.79417
				√					0.86012	0.93069	0.85267	0.79701
					√				0.87348	0.93512	0.87255	0.81277
						√			0.86757	0.93601	0.86942	0.79727
							√		0.87384	0.93521	0.87415	0.81215
								√	0.85859	0.92760	0.85984	0.78833
√			√						0.87125	0.93441	0.86729	0.81206
√				√					0.86832	0.93505	0.86264	0.80727
√					√				0.87326	0.93556	**0.87481**	0.80942
√							√		0.87026	0.93581	0.86720	0.80778
√								√	0.87213	0.93638	0.87206	0.80796
					√		√		**0.87476**	**0.93757**	0.87232	**0.81438**
							√	√	0.87445	0.93742	0.87164	0.81428
					√			√	0.87430	0.93730	0.87145	0.81416

## Data Availability

The datasets used and/or analyzed during the current study are available from the public dataset.
